# Iron biofortification of rice using different transgenic approaches

**DOI:** 10.1186/1939-8433-6-40

**Published:** 2013-12-19

**Authors:** Hiroshi Masuda, May Sann Aung, Naoko K Nishizawa

**Affiliations:** 1 Research Institute for Bioresources and Biotechnology, Ishikawa Prefectural University, 1-308 Suematsu, Nonoichi, Ishikawa 921-8836, Japan; 2 Graduate School of Agricultural and Life Sciences, The University of Tokyo, 1-1-1 Yayoi, Bunkyo-ku, Tokyo 113-8657, Japan

**Keywords:** Biofortification, Iron, Zinc, Transgenic rice, Nicotianamine, YSL, Ferritin, IDS3, Mugineic acids, Anemia

## Abstract

More than 2 billion people suffer from iron (Fe) deficiency, and developing crop cultivars with an increased concentration of micronutrients (biofortification) can address this problem. In this review, we describe seven transgenic approaches, and combinations thereof, that can be used to increase the concentration of Fe in rice seeds. The first approach is to enhance the Fe storage capacity of grains through expression of the Fe storage protein ferritin under the control of endosperm-specific promoters. Using this approach, the concentration of Fe in the seeds of transformants was increased by approximately 2-fold in polished seeds. The second approach is to enhance Fe translocation by overproducing the natural metal chelator nicotianamine; using this approach, the Fe concentration was increased by up to 3-fold in polished seeds. The third approach is to enhance Fe influx to the endosperm by expressing the Fe(II)-nicotianamine transporter gene *OsYSL2* under the control of an endosperm-specific promoter and sucrose transporter promoter, which increased the Fe concentration by up to 4-fold in polished seeds. The fourth approach is introduction of the barley mugineic acid synthesis gene *IDS3* to enhance Fe uptake and translocation within plants, which resulted in a 1.4-fold increase in the Fe concentration in polished seeds during field cultivation. In addition to the above approaches, Fe-biofortified rice was produced using a combination of the first, second, and third approaches. The Fe concentration in greenhouse-grown T_2_ polished seeds was 6-fold higher and that in paddy field-grown T_3_ polished seeds was 4.4-fold higher than in non-transgenic seeds without any reduction in yield. When the first and fourth approaches were combined, the Fe concentration was greater than that achieved by introducing only the *ferritin* gene, and Fe-deficiency tolerance was observed. With respect to Fe biofortification, the introduction of multiple Fe homeostasis genes is more effective than the introduction of individual genes. Moreover, three additional approaches, i.e., overexpression of the Fe transporter gene *OsIRT1* or *OsYSL15*, overexpression of the Fe deficiency-inducible bHLH transcription factor *OsIRO2*, and knockdown of the vacuolar Fe transporter gene *OsVIT1* or *OsVIT2*, may be useful to further increase the Fe concentration of seeds.

## Introduction

Iron (Fe) is an essential micronutrient for most organisms, including all plants and animals. Fe deficiency is one of the most prevalent micronutrient deficiencies globally, affecting an estimated two billion people (Stoltzfus et al. 1998) and causing 0.8 million deaths annually worldwide (WHO 2002). Fe deficiency is ranked sixth among the risk factors for death and disability in developing countries with high mortality rates (WHO 2002).

There are three basic approaches to alleviate micronutrient deficiencies: micronutrient supplementation, food fortification, and biofortification. Traditional public health interventions, including nutritional supplementation and industrial food fortification programs, have reduced micronutrient deficiencies worldwide. However, these interventions require infrastructure to produce micronutrient supplements or food fortifications, as well as purchasing power or access to markets and health care systems for their success, which are often not available to individuals living in remote rural areas (Mayer et al. 2008). These projects required continuous costs and are difficult to perform in developing countries. In contrast, biofortification (i.e., increasing the bioavailable concentration of essential elements in edible portions of crop plants through conventional breeding or genetic engineering) does not require specific processing after harvesting or a dedicated infrastructure (Grusak and DellaPenna 1999; Mayer et al. 2008). Therefore, biofortification is advantageous for both individuals and governments, as it is inexpensive and sustainable (Mayer et al. 2008). This can complement current efforts of nutritional supplementation and food fortification to address micronutrient deficiencies. Moreover, biofortification is beneficial for individuals who find it difficult to change their dietary habits owing to financial, cultural, regional or religious restrictions.

Rice is a particularly suitable target for biofortification because Fe-deficiency anemia is a serious problem in developing countries where rice is a major staple crop (WHO 2002; Juliano 1993). Brown rice is rich in mineral value. However, rice is mostly consumed in the polished form (the endosperm tissue), which contains low mineral levels (Grusak and Cakmak 2005). Biofortification can be applied to increase the Fe concentrations in polished seeds and achieve the target Fe demand for human nutrition.

**Figure 1:**
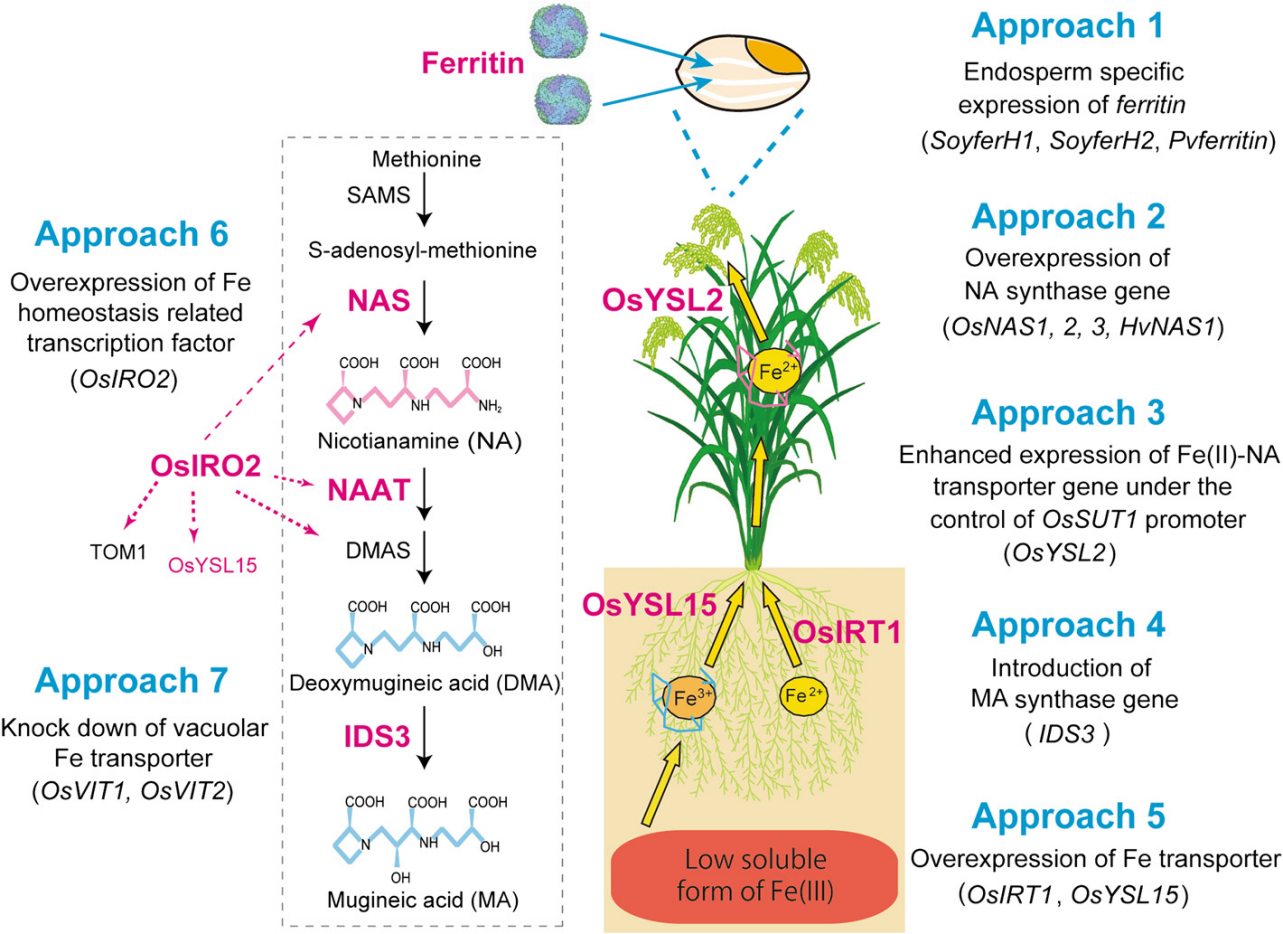
**Seven transgenic approaches to Fe biofortification of rice.** The pathway inside the gray dashed-line rectangle shows the biosynthetic pathway for mugineic acid family phytosiderophores (MAs) in graminaceous plants. SAMS, S-Adenosyl-methionine synthase; NAS, NA synthase; NAAT, NA aminotransferase; DMA, 2′-deoxymugineic acid; DMAS, DMA synthase; IDS3, MA synthase (dioxygenase that catalyzes the hydroxylation of DMA and epiHDMA at the 2′ position); Ferritin, iron storage protein; OsYSL2, Fe(II)-NA and Mn(II)-NA transporter; OsIRO2, Fe deficiency-inducible bHLH transcription factor related to Fe homeostasis in rice; OsIRT1, ferric transporter; OsYSL15, Fe(III)-DMA transporter; TOM1, MA transporter. Rice lacks the two dioxygenase genes (*IDS2* and *IDS3*) and secretes only DMA. **Approach 1**: Enhancing Fe accumulation in seeds by introducing the Fe storage protein, ferritin gene, *SoyferH1*, *SoyferH2* or *Pvferritin,* under the control of endosperm-specific promoters. **Approach 2**: Enhancing Fe transport within the plant body by the overexpression of *NAS*. **Approach 3**: Enhancing Fe influx to seeds by expression of the Fe(II)-NA transporter gene *OsYSL2* under the control of the *OsSUT1* promoter. **Approach 4**: Enhancing Fe uptake and translocation by introduction of the phytosiderophore synthase gene *IDS3*. **Approach 5**: Enhanced Fe uptake from soil by overexpression of the Fe transporter gene *OsIRT1* or *OsYSL15*. **Approach 6**: Enhanced Fe uptake and translocation by overexpression of the *OsIRO2* gene. **Approach 7**: Enhanced Fe translocation from flag leaves to seeds by knockdown of the vacuolar Fe transporter gene *OsVIT1* or *OsVIT2*. The ferritin image was kindly provided by Dr. David S. Goodsell (Scripps Research Institute, La Jolla, CA, USA) and the RCSB PDB.

Among the methods available for Fe biofortification of rice, transgenic methods can most efficiently increase Fe concentration in rice seeds. In this report, we describe seven recently reported transgenic approaches used to increase the Fe concentration of rice seeds (Figure 1, Table 1), and we propose some additional prospective target genes for the Fe biofortification of rice.

**Table 1:** Approaches of Fe biofortification of rice: single transgenic approaches

**Approach**	**Introduced genes**	**Rice cultivar**	**Cultivation condition**	**Fold increase in Fe concentration compared to non-transgenic rice**^**a**^	**References**
**Approach 1**	*OsGluB1* pro*-SoyferH1*	*Japonica* cv. Kitaake	Soil cultivation in greenhouse	2 fold (polished seeds)	Goto et al. 1999
Enhancement of Fe storage in rice seeds by *ferritin*					
				3 fold (brown seeds)	
	*OsGluB1* pro- *SoyferH1*^b^	*Japonica* cv. Kitaake	Soil cultivation in greenhouse	1.5 fold (brown seeds)	Qu et al. 2005
	*OsGlb1* pro-*SoyferH1*^b^				
	*OsGluB1* pro-*SoyferH1*	*Japonica* cv. Taipei 309	Soil cultivation in greenhouse	2.2 fold (brown seeds)	Lucca et al. 2002
	*OsGluB1* pro-*SoyferH1*	*Indica* cv. IR68144	Soil cultivation in greenhouse	3.7 fold (polished seeds)	Vasconcelos et al. 2003
		(High Fe breeder line)			
	*OsGluA2* pro-*OsFer2*	*Indica* cv. Pusa-Sugandh II (Aromatic rice)	Soil cultivation in greenhouse	2.1 fold (polished seeds)	Paul et al. 2012
**Approach 2**	*OsActin1* pro*-HvNAS1*^c^	*Japonica* cv. Tsukinohikari	Soil cultivation in greenhouse	2 fold (polished seeds)	Masuda et al. 2009
Enhancement of Fe translocation by overexpression of *NAS*					
	*35S* pro-*HvNAS1*^c^				
	Activation tag line of *OsNAS3*	*Japonica* cv. Dongjin	Soil culture in greenhouse	3 fold (polished seeds)	Lee et al. 2009c
	*35S* pro- *OsNAS1, 2, 3*	*Japonica* cv. Nipponbare	Soil cultivation in greenhouse	4 fold (polished seeds)	Johnson et al. 2011
**Approach 3**	*OsSUT1* pro-*OsYSL2*	*Japonica* cv. Tsukinohikari	Soil cultivation in greenhouse	4 fold (polished seeds)	Ishimaru et al. 2010
Enhancement of Fe transportation by Fe transporter					
**Approach 4**	Barley *IDS3* genome fragment	*Japonica* cv. Tsukinohikari	Andosol soil in paddy field	1.4 fold (polished seeds) 1.3 fold (brown seeds)	Masuda et al. 2008
Enhancement of Fe uptake and translocation by *IDS3* gene					
			Calcareous soil in paddy field	1.3 fold (brown seeds)	Suzuki et al. 2008
**Approach 5**	*Ubiquitin* pro-*OsIRT1*	*Japonica* cv. Dongjin	Paddy field	1.7 fold (leaves)	Lee et al.2009a
Overexpression of Fe transporter				1.1 fold (brown seeds)	
	*OsActin1* pro-*OsYSL15*	*Japonica* cv. Dongjin	Paddy field	1.3 fold (brown seeds)	Lee et al. 2009b
**Approach 6**	*35S* pro-*OsIRO2*	*Japonica* cv. Tsukinohikari	Calcareous soil in greenhouse	3 fold (brown seeds)	Ogo et al. 2011
Overexpression of transcription factor					
**Approach 7**	*OsVIT1* or *OsVIT2* T-DNA insertion mutant lines	*Japonica* cv. Zhonghua11 *Japonica* cv. Dongjin	Hydroponic culture	1.4 fold (brown seeds)	Zhang et al. 2012
Knockdown of *OsVITs* genes					
			Paddy field	1.4 fold (brown seeds)	
	*OsVIT2* T-DNA insertion mutant line	*Japonica* cv. Dongjin	Soil cultivation in greenhouse	1.3 fold (brown seeds)	Bashir et al. 2013
				1.8 fold (polished seeds)	

^a^The tissue name written in Parentheses is the rice tissue where Fe concentration was increased. ^b^They introduced these two genes into same transgenic lines. ^c^ These two genes were introduced separately into rice and they analyzed these two types of transgenic lines.

## Review

### Approach 1: Enhancing Fe accumulation in seeds by expression of the Fe storage protein, ferritin gene, *SoyferH1* and *SoyferH2,* under the control of endosperm-specific promoters

The first approach to increasing Fe concentration in rice seeds involves enhancing Fe accumulation in rice seeds by expressing the *ferritin* gene under the control of endosperm-specific promoters (Figure 1; Approach 1). Ferritin is a ubiquitous Fe storage protein that sequesters as many as 4,000 Fe atoms in a complex (Theil 2003). The Fe stored in soybean ferritin is readily absorbed by the human gastrointestinal tract (Lonnerdal 2009). The molecular mechanism underlying the uptake of Fe stored in food ferritin to the human body has been revealed (Theil 2011). Thus, Fe stored in ferritin is an important source for humans to use to avoid Fe deficiency (Theil et al. 2012).

Goto et al. (1999) generated transgenic rice plants expressing the soybean *ferritin* gene *SoyferH1* in endosperms using the rice endosperm-specific, 1.3-kb *OsGluB1* promoter. Transformants showed 3-fold higher levels of Fe accumulation in brown seeds (Table 1). Additionally, the concentration of Fe in the endosperm was increased 2-fold. This endosperm-specific expression of *ferritin* has been used to generate the following increases in Fe concentration in rice plants: a 2.2-fold increase in brown seeds of transgenic *japonica* cv. Taipei 309 (Lucca et al. 2002), a 3.7-fold increase in polished seeds of *indica* cv. IR68144 (Vasconcelos et al. 2003), and a 2.1-fold increase in polished seeds of *indica* cv. Pusa Sugandhi II (Paul et al. 2012) (Table 1).

Furthermore, Qu et al. (2005) expressed the *SoyferH1* gene under the control of two endosperm specific promoters, *OsGlb1* promoter and 1.3-kb *OsGluB1* promoter, to increase further the concentration of Fe in the seed. However, using multiple promoters to increase the level of *ferritin* expression in rice seeds did not significantly increase the Fe concentration compared to expression driven by a single endosperm-specific promoter (Table 1; Qu et al. 2005).

In our previous study, the Fe concentration in brown seeds was not increased in transgenic rice introduced only *ferritin* compared to non transgenic line (Masuda et al. 2013). Moreover, the introduction of *ferritin* gene alone produced symptoms of Fe deficiency in the leaves of transgenic plants (Qu et al. 2005; Masuda et al. 2013). Thus, improving *ferritin* expression may not be sufficient to increase Fe concentration in rice grains.

Generally, Fe translocation ability to endosperm is low in rice. In fact, the Fe content of endosperm is significantly lower than in other rice tissue, including leaves, stems, roots, husk, or the aleurone layer. 0.5% of total Fe content in aerial parts of plant body was found in polished seeds (endosperm) of Tsukinohikari rice variety, despite 3.6% in brown seeds, 3.4% in husk, 2.4% in rachis and 90.6% in straw, respectively (Masuda et al. 2008). Rice plants uptake certain amount of Fe, but the plant may not translocate it to endosperm positively. A strict regulation system may exist to control Fe translocation into endosperm in rice plants. This may explain why this approach does not significantly increase the Fe concentration in polished seeds.

Therefore, in addition to increased Fe storage in seeds, enhanced uptake of Fe from the soil and enhanced translocation of Fe within the plant body are required to improve further the Fe biofortification of rice seeds. Thus, the following approaches (from approach 2 to 7) are considered as ways to increase the Fe concentration of the rice endosperm (Figure 1).

### Approach 2: Enhancing Fe transport within the plant body by overexpression of the nicotianamine synthase gene *NAS*

The uptake, translocation, and homeostasis of Fe in rice are beginning to be understood at the molecular level, and many of the associated genes have been identified (Bashir et al. 2010; Kobayashi et al. 2012). As a second approach to increase Fe concentrations in rice, we overexpressed the nicotianamine (NA) synthase gene (*NAS*) (Figure 1; Approach 2).

NA, which is a chelator of metal cations such as Fe(II) and zinc (Zn)(II), is biosynthesized from methionine *via* S-adenosyl methionine synthase (SAMS) and NAS (Figure 1; Higuchi et al. 1994). All higher plants synthesize and utilize NA for the internal transport of Fe and other metals (Hell and Stephan 2003; Takahashi et al. 2003). Indeed, the NA-defective tomato mutant *chloronerva* (Rudolph et al. 1985) has a phenotype that is indicative of Fe deficiency (Pich and Scholz 1996;Stephan et al. 1996). Takahashi et al. (2003) produced NA-deficient transgenic tobacco plants by constitutively expressing the barley NA aminotransferase (NAAT) gene, *HvNAAT-A*. The obtained transformants showed interveinal chlorosis in the young leaves, and the Fe and Zn concentrations in the leaves and flowers decreased as a result of disrupted internal metal transport. Conversely, overexpression of the barley *NAS* gene *HvNAS1* increased the concentrations of Fe and Zn in the leaves, flowers, and seeds of tobacco plants (Takahashi et al. 2003).

Rice has three *NAS* genes; *OsNAS1*, *OsNAS2*, and *OsNAS3.* Although these genes are differentially regulated by Fe, all of them are expressed in cells involved in the long-distance transport of Fe (Inoue et al. 2003). These results suggest that NAS and NA play important roles in the long-distance transport of Fe in rice, in addition to their roles in phytosiderophore synthesis.

Higuchi et al. (2001a) produced transgenic rice expressing *HvNAS1* under the control of the cauliflower mosaic virus constitutive *CaMV35S* promoter. NA concentration in plant shoot increased 3-fold in this transgenic rice. Based on these results, we hypothesized that enhancing the expression of *NAS* could increase the NA concentration within the plant body and, as a consequence, increase the Fe concentration in seeds. Therefore, we produced transgenic rice overexpressing *HvNAS1* under the control of the *OsActin1* promoter or *35S* promoter (Masuda et al. 2009). *HvNAS1*-overexpressing transgenic rice showed increased *HvNAS1* expression and increased endogenous NA levels in the shoots and seeds by 5 to 10-fold (Masuda et al. 2009). The Fe and Zn concentrations in the T_1_ polished seeds from the transgenic plants increased more than 3-fold and 2-fold, respectively. The Fe concentrations also increased 2-fold in the T_2_ polished seeds (Table 1). Lee et al. (2009c) and Johnson et al. (2011) showed that *NAS* overexpression increased the Fe concentration in polished rice seeds by 3–4-fold (Table 1). Thus, Fe transport within the plant body, including the phloem, may be improved by *NAS* overexpression (Masuda et al. 2009; Lee et al. 2009c; Johnson et al. 2011). Moreover, Lee et al. (2009c) reported that Fe-biofortified rice overexpressing *OsNAS3* by virtue of an enhancer tag mitigated Fe-deficiency anemia in mice to a greater degree than non-transgenic rice seeds.

Importantly, not only the NA content but also the deoxymugineic acid (DMA) content was increased by overexpressing *HvNAS1* in rice (Masuda et al. 2009, Lee et al. 2009c). In graminaceous plants, including rice, DMA is synthesized from NA by NAAT and DMA synthase (DMAS) (Figure 1) (Takahashi et al. 1999; Bashir et al. 2006; Inoue et al. 2008). Overexpression of *AtNAS1* together with endosperm expression of *Pvferritin* in rice enhanced the expression of *OsSAMS2*, *OsNAS1*, *OsNAS3* and *OsDMAS1* in roots (Wang et al. 2013). Enhanced expression of endogenous NAS and DMAS might contribute to DMA production in rice. In addition, DMA concentration in rice xylem sap was increased in Fe deficient rice (Kakei et al. 2009). This suggested that DMA contributes to the transport of Fe from root to shoot through xylem.

Moreover, OsYSL15, a Fe(III)-DMA transporter, is expressed in the root epidermis and stele, and contributes to the internal translocation of Fe (Inoue et al. 2009). Aoyama et al. (2009) showed that OsYSL18, a Fe(III)-DMA transporter, was expressed in reproductive organs and phloem of lamina joints. Kakei et al. (2012) also showed that OsYSL16, another Fe(III)-DMA transporter, was expressed in both root epidermis and vascular bundles of whole plants. Furthermore, Nishiyama et al. (2012) described that Fe(III)-DMA complexes were detected as low-molecular-weight chemical forms of Fe in rice phloem sap, and DMA may play a role in long-distance Fe transport in phloem sap. These results suggest that, similar to NA, DMA is involved in Fe distribution from roots to seeds in rice plant. Thus, the overproduction of NA and subsequent increase in DMA content may enhance the translocation of Fe and Zn into rice grains (Masuda et al. 2009). Moreover, an increase in the DMA content of rice may increase DMA secretion from the roots and contribute to enhancing the uptake of Fe from the soil. Therefore, increasing the NA and DMA concentrations by enhancing *NAS* expression may increase the levels of Fe and Zn in rice grains.

### Approach 3: Enhancing Fe influx to seeds by expressing the Fe (II)-NA transporter gene *OsYSL2* under the control of the *OsSUT1* promoter

Manipulating the expression of membrane transporters can increase Fe concentrations in crops (Schroeder et al. 2013). Thus, the third approach to increase Fe concentrations in rice was to enhance Fe influx to seeds by expressing the Fe(II)-NA transporter gene *OsYSL2* (Figure 1; Approach 3). Koike et al. (2004) identified the rice *OsYSL2* gene, which is preferentially expressed in leaf phloem cells, the vascular bundles of flowers and developing seeds, suggesting a role in internal Fe transport. The Fe concentration in *OsYSL2* knockdown rice was 18% lower in brown seeds and 39% lower in polished seeds compared with non-transgenic rice (Ishimaru et al. 2010). Thus, OsYSL2 plays an important role in the transport of Fe to rice seeds.

Therefore, we hypothesized that enhanced expression of *OsYSL2* could increase the Fe concentration in rice seeds. However, overexpression of *OsYSL2* under the control of the *CaMV35S* promoter did not increase the concentration of Fe in the seeds, although it did increase the Fe concentration in the roots (Ishimaru et al. 2010). Constitutive expression of *OsYSL2* may disrupt Fe translocation in rice.

A rice sucrose transporter, *OsSUT1,* was expressed in companion cells of the phloem in flag leaves and the rachis (Scofield et al. 2007). *OsSUT1* was also strongly expressed in the panicle and immature seeds during seed maturation (Aoki et al. 2003). Moreover, *OsSUT1* antisense rice showed significant reductions in sucrose uptake and filling rates in seeds (Ishimaru et al. 2001). OsSUT1 is a key transporter of sucrose from the phloem to seeds. Therefore, the *OsSUT1* promoter is a suitable promoter to control seed metal concentrations in rice. Takahashi et al. (2012) also expressed the Zn and Cd transporter *OsHMA2* under the control of the *OsSUT1* promoter, and successfully increased Zn concentration by 20% and decreased Cd concentration by 50% in brown seeds compared to non-transgenic rice.

Therefore, to effectively target *OsYSL2* expression, we engineered a construct in which *OsYSL2* expression was controlled by *OsSUT1* promoter, and introduced the construct into rice to enhance Fe(II)-NA translocation into seeds (Ishimaru et al. 2010). We found that the enhanced *OsYSL2* expression under the control of the *OsSUT1* promoter increased the Fe concentration up to 4-fold in polished rice seeds (Table 1) (Ishimaru et al. 2010). Thus, expression of the Fe(II)-NA transporter gene *OsYSL2* under the control of the *OsSUT1* promoter is one of the remarkable approaches to increase the Fe concentration in rice seeds.

### Combination of Approaches 1–3

Wirth et al. (2009) combined the first and second approaches by introducing the *CaMV35S* promoter-*AtNAS1* and *Globulin* promoter-*Pvferritin* genes into the *Japonica* cv. Taipei 309 rice variety (Table 2). They also introduced the *Globulin* promoter-*Afphytase* gene cassette to reduce the phytate content and improve Fe bioavailability in rice seeds. This rice, named NFP rice, showed increased Fe concentrations by up to 6-fold in hydroponic cultures under various Fe nutrient conditions (Table 2).

**Table 2:** Approaches of Fe biofortification of rice: multi transgenic approaches

**Approach**	**Introduced genes**^**a**^	**Rice cultivar**	**Cultivation**	**Fold increase in Fe concentration compared to non-transgenic rice**^**b**^**.**	**References**
			**condition**		
**Combination of approaches 1 and 2**	*OsGlb* pro-*Pvferritin*	*Japonica* cv. Taipei 309	Hydroponic culture	6 fold (polished seeds)	Wirth et al. 2009
	*35S* pro-*AtNAS1*				
	*OsGlb* pro-*Afphytase*				
**Combination of approaches 1, 2 and 3**	*OsGluB1* pro-*SoyferH2*	*Japonica* cv. Tsukinohikari	Soil cultivation in greenhouse	6 fold (polished seeds)	Masuda et al. 2012
	*OsGlb1* pro-*SoyferH2*				
	*OsActin1* pro-*HvNAS1*		Paddy field	4.4 fold (polished seeds)	
	*OsSUT1* pro-*OsYSL2*				
	*OsGlb1* pro-*OsYSL2*				
	*OsGluB1* pro-*SoyferH2*	*Tropical Japonica* cv. Paw San Yin (Myanmar High Quality Rice)	Soil cultivation in greenhouse	3.4 fold (polished seeds)	Aung et al. 2013
	*OsGlb1* pro-*SoyferH2*				
	*OsActin1* pro-*HvNAS1*				
	*OsSUT1* pro-*OsYSL2*				
	*OsGlb1* pro-*OsYSL2*				
**Combination of approaches 1 and 4**	*OsGluB1* pro-*SoyferH2*	*Japonica* cv. Tsukinohikari	Normal soil in greenhouse	4 fold (polished seeds)	Masuda et al. 2013
	*OsGlb1* pro-*SoyferH2*				
	*HvNAS1*, *HvNAAT-A,-B* and *IDS3* genome fragments		Calcareous soil in greenhouse	2.5 fold (polished seeds)	

^a^These gene expression cassettes were introduced concomitantly. ^b.^The tissue name written in parentheses is the rice tissue where Fe concentration was increased.

**Figure 2:**
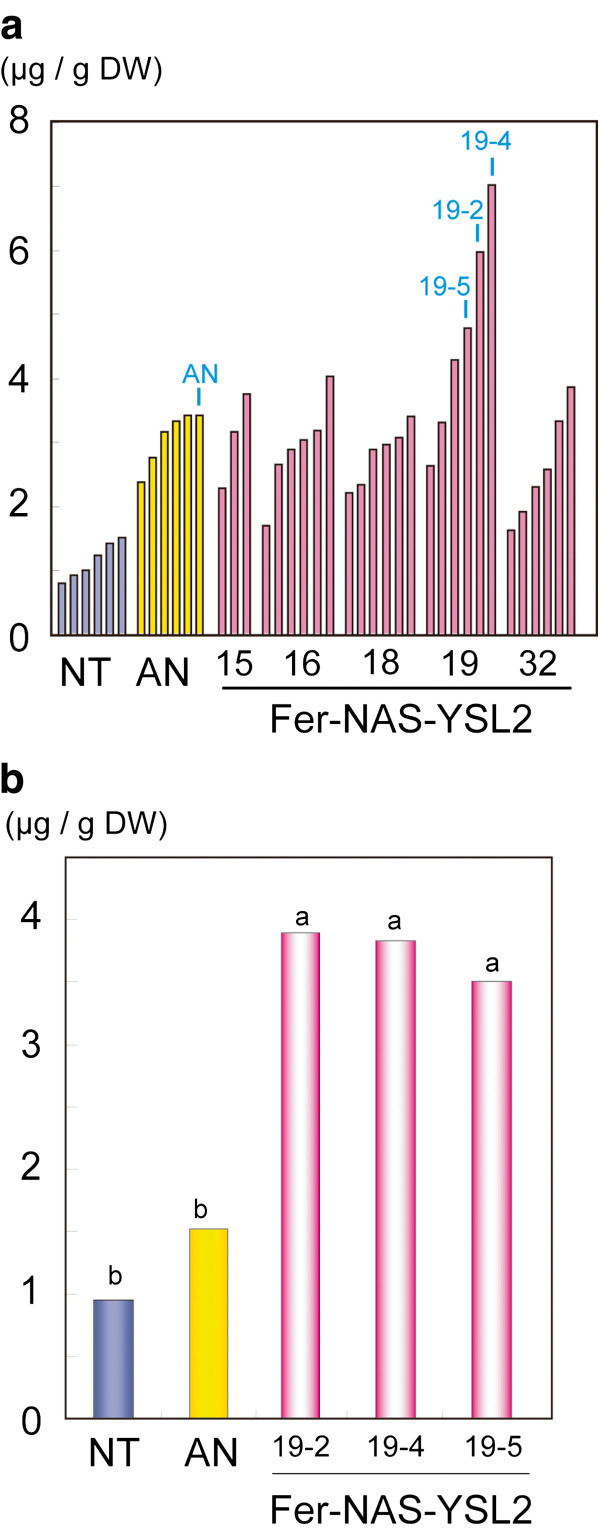
**Fe concentration in the Fer-NAS-YSL2 lines (Masuda et al.**^2012^**). a**; The Fe concentrations in T_2_ polished rice seeds (*Oryza sativa* cv. Tsukinohikari). Bars represent the Fe concentrations in polished seeds obtained from individual transgenic or non-transgenic plants. The numbers indicate the line numbers of the independent T_1_ lines. The arrows and numbers above the bars show the lines that contained high levels of Fe, which are shown in **b** for the subsequent generation. **b**; Fe concentrations in the T_3_ polished rice seeds (*Oryza sativa* cv. Tsukinohikari) harvested from the paddy field. ANOVA with the Tukey–Kramer HSD test was used for each four-block dataset (n = 4). The letters above the bars indicate significant differences (*P* < 0.05). NT, Non-transgenic rice; AN, *OsActin1* promoter–*HvNAS1* transgenic rice line No. 8 (Masuda et al. 2009); Fer-NAS-YSL2, transgenic rice lines that carry the *OsGlb1* promoter–*SoyferH2*, *OsGluB1* promoter–*SoyferH2*, *OsSUT1* promoter–*OsYSL2*, *OsGlb1* promoter–*OsYSL2*, and *OsActin1* promoter–*HvNAS1* (Masuda et al. 2012).

We generated Fe-biofortified rice using a combination of the first, second, and third approaches (Figure 1). We produced 45 independent transgenic rice lines (*Japonica* cv. Tsukinohikari; Fer-NAS-YSL2 lines), which included the *OsGluB1* promoter–*SoyferH2*, *OsGlb1* promoter–*SoyferH2*, *OsActin1* promoter–*HvNAS1*, *OsSUT1* promoter–*OsYSL2*, and the *OsGlb1* promoter–*OsYSL2* gene cassettes (Table 2) (Masuda et al. 2012). The Fe concentrations in the T_2_ seeds were increased up to 6-fold in the Fer-NAS-YSL2 lines and 3-fold in the *OsActin1* promoter-*HvNAS1* lines compared with the non-transgenic line under soil cultivation in a greenhouse (Figure 2a). For practical applications, we explored whether the Fer-NAS-YSL2 lines set seeds with increased Fe concentrations under actual paddy field conditions. To accomplish this, selected T_2_ lines were cultivated in an isolated paddy field. We found that the Fe concentration in the paddy field-grown T_3_ polished seeds was 4.4-fold higher than in non-transgenic seeds (Figure 2b), without any loss of yield. Moreover, the transgenic seeds accumulated Zn at levels up to 1.6-fold higher in the field. Our results demonstrated that the introduction of multiple Fe homeostasis genes is more effective at achieving Fe biofortification than the introduction of individual genes under soil cultivation in greenhouse and paddy field conditions.

Fe concentration was increased significantly in Fer-NAS-YSL2 rice (Masuda et al. 2012) because the introduced genes, *ferritin*, *HvNAS1* and *OsYSL2,* functioned synergistically as follows: Overexpression of the *NAS* gene increased the NA and DMA concentrations in the plant body. Abundant NA and DMA facilitated the formation of Fe(II)–NA or Fe(III)–DMA. Additionally, Fe(II) or Fe(III) transport in the plant body, including in the phloem, was improved by *NAS* overexpression. For effective translocation of enhanced Fe(II)–NA in phloem sap, we engineered the Fe(II)–NA transporter *OsYSL2* to be under the control of the *OsSUT1* promoter. Additionally, *OsYSL2* expressed in endosperm cells under the control of the *OsGlb1* promoter may enhance transport of Fe(II)–NA into endosperm cells. Moreover, *ferritin* expressed under the control of the *OsGlb1* promoter and the *OsGluB1* promoter accumulated Fe in seed endosperm cells. As a result, Fe concentration in the polished seeds of Fer-NAS-YSL2 rice was markedly elevated than Fe concentration achieved by a single-transgenic approach (Masuda et al. 2012).

Fe deficiency anemia is prevalent in many populations in Myanmar (MOH 2003). Myanmar has one of the highest per capita rice consumption rates (average, 578 g per day) globally (Maclean et al. 2002). Thus, Myanmar rice is a suitable target for Fe biofortification. Therefore, we introduced this Fer-NAS-YSL2 gene into the Paw San Yin variety, which is a popular high-quality rice variety in Myanmar (Aung et al. 2013). Paw San Yin transgenic rice was produced successfully, and the concentration of Fe in the Paw San Yin Fer-NAS-YSL2 line increased 3.4-fold in polished seeds, which was close to the dietary target level for people in Myanmar (Table 2) (Aung et al. 2013).

### Approach 4: Enhancing Fe uptake and translocation by introducing the phytosiderophore synthase gene *IDS3*

Graminaceous plants secrete mugineic acid family phytosiderophores (MAs), which are natural Fe(III) chelators crucial to take up Fe from the rhizosphere (Takagi 1976; Mihashi and Mori 1989). Methionine has been identified as the precursor of MAs (Figure 1) (Mori and Nishizawa 1987). In graminaceous plants including rice, DMA is synthesized from NA by NAAT and DMAS (Figure 1), and in barley and some graminaceous species, other types of MAs are synthesized from DMA by Fe deficiency-specific clones no. 2 (IDS2) and no. 3 (IDS3, mugineic acid synthase) (Nakanishi et al. 2000; Kobayashi et al. 2001). Graminaceous plant roots secrets MAs, which chelate insoluble Fe(III) in the rhizosphere. The resulting Fe(III)-MAs complexes are absorbed into the roots *via* the transporter YS1 or YSL (Curie et al. 2001). In rice, DMA is secreted from roots by the transporter of mugineic acid family phytosiderophores 1 (TOM1) in rice (Nozoye et al. 2011), and Fe(III)-DMA complexes are thought to be absorbed *via* the OsYSL15 transporter (Inoue et al. 2009; Lee et al. 2009b).

Among graminaceous plants, barley is highly tolerant to Fe deficiency and possesses a series of biosynthetic genes for MAs, including *HvNAS1*, *HvNAAT-A*, *HvNAAT-B*, *HvDMAS1*, *IDS2*, and *IDS3*, the expression levels of which are up-regulated in Fe-deficient barley roots (Higuchi et al. 1999; Takahashi et al. 1999; Nakanishi et al. 2000; Bashir et al. 2006). In contrast, rice lacks *IDS2* and *IDS3* and secretes only DMA. This is thought to be one of the reasons why barley has a higher tolerance than rice to Fe deficiency (Kobayashi et al. 2001).

Thus, a fourth approach to Fe biofortification involves enhancing Fe uptake and translocation by introducing genes responsible for biosynthesis of MAs (Figure 1; Approach 4). Three transgenic rice lines, which were transformed with the barley genome fragments involving mugineic acid synthase genes, *HvNAS1* or *HvNAS1* and *HvNAAT-A*,*-B* or *IDS3,* were produced (Higuchi et al. 2001b; Kobayashi et al. 2001; Masuda et al. 2008; Suzuki et al. 2008). The Fe concentrations in the seeds of transgenic lines were analyzed after cultivation in the paddy field in Fe-sufficient (andosol) or low Fe-available (calcareous) soil (Masuda et al. 2008; Suzuki et al. 2008). The *IDS3* rice lines produced Fe concentrations that were 1.4-fold and 1.3-fold higher in the polished and brown seeds than in non-transgenic rice respectively after cultivation in Fe-sufficient soil (Table 1) (Masuda et al. 2008). Fe concentrations of the *IDS3* rice lines were also 1.3-fold higher in the brown seeds in low Fe-available soil (Table 1) (Suzuki et al. 2008).

In addition to DMA, the introduction of *IDS3* conferred upon the rice the ability to secrete MA (Kobayashi et al. 2001). As MA showed greater Fe(III)-complex stability than DMA at a slightly acidic pH (von Wirén et al. 2000), the production of MA *via IDS3* may be beneficial for Fe translocation in rice. Furthermore, since these transformants contained introduced barley genome fragments, expression of the genes responsible for the biosynthesis of MAs was regulated by their own promoters. In rice, these promoters induce expression in response to Fe deficiency in roots and leaves (Higuchi et al. 2001b; Kobayashi et al. 2001; Takahashi et al. 2001). Thus, these genes are likely expressed where and when the requirement for Fe is elevated.

### Combination of Approaches 1 and 4

We attempted to increase the Fe concentrations in rice seeds using a combination of the first and fourth approaches (Figure 1). We introduced the *SoyferH2* gene driven by two endosperm-specific promoters (the *OsGluB1* promoter and *OsGlb1* promoter), together with three fragments of the barley genome (5.9 kb of *HvNAS1*, 11 kb of *HvNAAT-A*,-*B*, and 6 kb of *IDS3*) to enhance MA production in rice plants (Table 2) (Masuda et al. 2013). Representative lines were selected and grown in commercially supplied soil (Fe-sufficient condition) or calcareous soil (low Fe-available condition). Lines expressing both *ferritin* and the MA biosynthetic genes showed signs of tolerance to Fe deficiency in the calcareous soil. The concentrations of Fe in the T_3_ polished seeds were increased by 4- and 2.5-fold compared with the levels in non-transgenic lines grown in normal soil and calcareous soil, respectively (Table 2). During calcareous soil cultivation, the Fe concentration in polished seeds was increased in Fer-NAS-NAAT-IDS3 lines, but not in the Ferritin-introduced line compared to non-transgenic lines. These results indicated that the concomitant introduction of the *ferritin* and MA biosynthetic genes effectively increased Fe levels in seeds without inducing Fe sensitivity under Fe-limited conditions.

### Approach 5: Enhanced Fe uptake from soil by overexpression of the Fe transporter gene *OsIRT1* or *OsYSL15*

Fe concentrations in rice seeds are generally low (Grusak and Cakmak 2005). Especially, rice is mainly consumed as polished seeds, and Fe levels are diminished by milling and polishing; thus, the remaining endosperm contains low Fe levels. In addition, within a rice-consuming country, rice consumption is lower in urban areas than in rural areas (Juliano, 1993). Therefore, a higher target Fe concentration in polished rice grain is desirable.

Rice consumption per capita per day ranges from 300 to 600 g in South East Asia and China (Maclean et al. 2002). The Fe requirement for adult females is 15–18 mg per day (Food and Nutrition Board 2001). Thus, the target Fe concentration in rice ranges from 7 to 14 ppm in the polished grain for individuals that consume 600 g or 300 g per day, respectively. This would account for ~25% of the daily Fe requirement. Pfeiffer and McClafferty (2007) also proposed similar target level for Fe biofortification.

Fe concentrations in various indica rice varieties harvested in the paddy field mostly ranged from 1 to 2 ppm (Aung et al., unpublished data). Therefore, the Fe concentration should be increased by 4–10-fold to reach the target levels. This target Fe requirement has not been achieved using approaches 1-4 alone, which resulted in a maximum 4-fold increase (Table 1). A combination of approaches 1-3 achieved a 6-fold increase (Table 2). However, this may not be sufficient in some cases. Thus, we proposed three additional approaches (approaches 5, 6 and 7) to further Fe biofortification in seeds. Approach 5 involves enhancing Fe uptake by overexpressing the Fe transporter (Figure 1; Approach 5).

Previous studies have reported increased concentrations of Fe in seeds after overexpressing the Fe transporter. Lee et al. (2009a) produced transgenic rice that expressed the rice ferric ion transporter gene *OsIRT1* under the control of the *Ubiquitin* promoter. This rice showed a 13% increase in Fe concentration in the brown seeds (Table 1), while the Fe concentration in the leaves increased 1.7-fold. The authors suggested that *OsIRT1* could be used to enhance Fe levels in rice grains. Next, Lee et al. (2009b) reported that *OsYSL15* overexpression using the *OsActin1* promoter increased the concentration of Fe in brown seeds by approximately 1.3-fold compared with non-transgenic rice (Table 1). In addition, Gómez-Galera et al. (2012) produced transgenic rice that overexpressed the barley Fe(III)-MA transporter gene *HvYS1* under the control of the *CaMV35S* promoter. The concentration of Fe in the transgenic leaves was 1.5-fold higher than in the non-transgenic leaves. In this previous study, it was suggested that Fe uptake from the rhizosphere could be enhanced by expressing *HvYS1*. Although overexpression of *OsIRT1*, *OsYSL15* or *HvYS1* increased the Fe concentrations in the leaves, it did not significantly increase Fe concentrations in the seeds (Table 1) (Lee et al. 2009a; Lee et al. 2009b; Gómez-Galera et al. 2012). Lee et al. (2009a) reported that plants overexpressing *OsIRT1* were shorter and had fewer tillers. As constitutive expression of *OsIRT1* may disturb metal homeostasis in rice, they suggested that targeted expression of *OsIRT1* using specific promoters might solve this problem.

Application of this approach 5 alone did not remarkably increase the Fe concentrations in seeds. However, it is possible that this approach in combination with other approaches (i.e., Approaches 1–3) could increase Fe levels.

### Approach 6: Enhanced Fe uptake and translocation by overexpression of the Fe homeostasis-related transcription factor OsIRO2

Ogo et al. (2006) identified a Fe-deficiency-inducible basic helix–loop–helix (bHLH) transcription factor, OsIRO2, in rice. OsIRO2 is responsible for regulation of the key genes involved in MAs-related Fe uptake; e.g., *OsNAS1, OsNAS2, OsNAAT1, OsDMAS1, TOM1*, and *OsYSL15* (Figure 1) (Ogo et al. 2007; Ogo et al. 2011). Ogo et al. (2007) introduced *OsIRO2* under the control of the CaMV*35S* promoter into rice plants. Rice that overexpressed *OsIRO2* secreted more DMA than non-transgenic rice, and exhibited enhanced Fe-deficiency tolerance in calcareous soils (Ogo et al. 2007; Ogo et al. 2011). Moreover, the concentration of Fe in the transgenic brown seeds was increased 3-fold when the transgenic rice was cultivated in calcareous soil (Table 1) (Ogo et al. 2011). Therefore, this approach can be used to increase the Fe concentrations in seeds in soils with low Fe availability.

### Approach 7: Enhanced Fe translocation from flag leaves to seeds by knockdown of the vacuolar Fe transporter gene *OsVIT1* or *OsVIT2*

Kim et al. (2006) have reported that the *Arabidopsis* vacuolar Fe transporter, VIT1, is highly expressed in developing seeds and transports Fe and manganese into the vacuole. Zhang et al. (2012) reported that disruption of the rice *VIT* orthologues (*OsVIT1* and *OsVIT2*) increased the Fe concentrations by 1.4-fold in brown seeds (Table 1) and decreased the Fe concentrations by 0.8-fold in the source organ flag leaves. A possible explanation for these results is that the *VIT* genes are highly expressed in rice flag leaves. Bashir et al. (2013) also reported that an *OsVIT2*-knockdown mutant showed 1.3-fold and 1.8-fold increases in Fe concentrations in the brown and polished rice seeds, respectively (Table 1). They suggested that disruption of OsVIT1 or OsVIT2 enhanced Fe translocation between the source and sink organs, and proposed this as a novel strategy for producing Fe-biofortified rice (Figure 1).

Zhang et al. (2012) and Bashir et al. (2013) reported that the Cd concentration was also increased in *VIT* knockdown rice. Therefore, this approach should be avoided in Cd-contaminated soils. Although further studies are required for this approach, likewise approach 5 and 6, approach 7 may also be applied in combination with other approaches to further increase Fe concentrations in polished seeds.

### Alternative strategies to increase Fe transport within rice plants, with the goal of increasing the Fe concentration in seeds

An alternative approach to achieving Fe biofortification involves enhancing Fe chelate export by the overexpression of genes for mugineic acid or protocatechuic acid exporter. The MA transporter gene, *TOM1,* is expressed in restricted regions of the exodermis of roots under Fe-sufficient conditions and throughout the roots under Fe-deficient conditions (Nozoye et al. 2011). *TOM1* is also expressed in the vascular bundles of the leaf sheaths and leaf phloem, the pollen, and the dorsal vascular bundle in developing seeds. Nozoye et al. (2011) produced rice that overexpressed *TOM1*; in this rice, DMA secretion was enhanced, the Fe concentration was increased 1.2-fold, the Zn concentration was increased 1.6-fold in the rice seeds, and tolerance to Fe deficiency was increased.

Plants secrete phenolics to absorb apoplasmic precipitated Fe, such as protocatechuic acid (PCA) (Cesco, et al. 2010). Ishimaru et al. (2011) identified a phenolics efflux transporter, PEZ1, in rice. PEZ1 localized to the plasma membrane and transported PCA when expressed in *Xenopus laevis* oocytes. PEZ1 is responsible for increasing the PCA concentration in the xylem sap, and is essential for the utilization of apoplasmic precipitated Fe in the stele. PEZ1 localized mainly to the stele of the roots. The concentration of Fe in the leaves of transgenic rice lines containing the *CaMV35S* promoter-*PEZ1* gene cassette was increased 3-fold. The concentration of Fe in the roots of this transgenic rice was also increased 2-fold due to the high solubilization level of apoplasmic precipitated Fe in the stele (Ishimaru et al. 2011). Consequently, the concentration of Fe in seeds may increase by overexpression of *PEZ1*.

Therefore, the seed Fe concentration may be increased through chelate export using the *TOM1* or *PEZ1* gene and a combination of the other approaches; e.g., endosperm-specific expression of the *ferritin* gene (Approach 1; Figure 1), overexpression of the *NAS* gene (Approach 2; Figure 1), or *OsYSL2* expression under the control of the *OsSUT1* promoter (Approach 3; Figure 1).

### Mining of high-Fe rice varieties or other target genes for Fe biofortification of rice

Increases in Fe concentrations using transgenic approaches are dependent on the cultivar. The target Fe concentration using transgenic approaches should be considered based on the Fe concentration level of the host rice variety. Based on this information, the required increase in Fe concentration can be calculated. Therefore, to produce rice lines with higher Fe concentrations, it is worthwhile to apply transgenic methods to original high-Fe rice varieties obtained through mining among extant rice varieties, or high-Fe rice lines produced by conventional breeding, or high-Fe mutant rice lines.

The mining of high-Fe rice varieties or the identification of novel target genes is important to Fe biofortification of rice. Anuradha et al. (2012) found seven quantitative trait loci (QTL) and selection markers related to the concentrations of Fe in rice seeds. They performed mapping of QTL using Madhukar × Swarna indica rice varieties and identified genes related to Fe homeostasis, such as *OsYSLs*, *OsNASs*, *OsNRAMP1*, *OsIRT1*, *OsZIPs* and *APRT*, as candidate genes that effect the concentration of Fe in seeds.

Sperotto et al. (2010) analyzed the gene expression profiles of 25 metal-related genes, including rice homologues of *YSL2*, *NRAMPs*, *ZIPs*, *IRT1*, *VIT1, NASs, FROs* and *NAC5,* in eight rice varieties with different Fe and Zn concentrations in the seeds. They also identified putative target genes that contribute to increasing the Fe and Zn concentrations in rice grains.

Jeng et al. (2012) discovered mutant lines that have higher Fe or Zn concentrations in polished seeds by searching among NaN_3_-induced mutant lines (*Oryza sativa* cv. IR64). Ruengphayak et al. (2012) screened 12,000 fast neutron-irradiated M_4_ mutant lines (*Oryza sativa* cv. Jao Hom Nin) and identified 76 mutant lines that contained higher Fe densities in the grains. Using these high-Fe mutant rice lines, it is possible to identify novel candidate genes to improve Fe biofortification of rice.

Some studies have been conducted to improve mineral nutrition in rice seeds through traditional breeding or marker-assisted breeding. IR68144 rice derived from conventional breeding method was shown to have 2-fold higher Fe concentrations in its seeds (Gregorio et al. 2000). This IR68144 rice has been shown to be superior to normal rice in improving the Fe status of women (Haas et al. 2005).

## Conclusions

We generated transgenic rice by introducing multiple genes, including *ferritin* under the control of endosperm-specific promoters, *NAS* overexpression, *OsSUT1* promoter-driven *OsYSL2* expression, and the barley *IDS3* genome fragment, and showed increased concentrations of bioavailable Fe. This technique could be applied to mitigate the global problem of Fe-deficiency anemia. However, further efforts in Fe biofortification of rice are required to increase further Fe concentrations in polished seeds and reach the recommended levels. Increasing the expression of *OsIRT1*, *OsYSL15,* and *OsIRO2*, or knockdown of *OsVIT1* or *OsVIT2* are the candidate approaches to improve Fe biofortification of rice seeds. Further attempts are required to evaluate high-Fe rice varieties or other target genes. A combination of these approaches will be beneficial to future Fe biofortification work.

## Competing interests

The authors declare that they have no competing interests.

## Authors’ contributions

The manuscript was written by HM and improved by MSA and NKN. All the authors read and approved the final manuscript.
